# PennPET Explorer: Design and Preliminary Performance of a Whole-Body Imager

**DOI:** 10.2967/jnumed.119.229997

**Published:** 2020-01

**Authors:** Joel S. Karp, Varsha Viswanath, Michael J. Geagan, Gerd Muehllehner, Austin R. Pantel, Michael J. Parma, Amy E. Perkins, Jeffrey P. Schmall, Matthew E. Werner, Margaret E. Daube-Witherspoon

**Affiliations:** 1 Department of Radiology, University of Pennsylvania, Philadelphia, Pennsylvania; 2 Department of Biomedical Engineering, University of Pennsylvania, Philadelphia, Pennsylvania; 3 KAGE Medical, Wayne, Pennsylvania; and; 4 Philips Healthcare, Highland Heights, Ohio

**Keywords:** PET, whole-body imager, NEMA performance

## Abstract

We report on the development of the PennPET Explorer whole-body imager. **Methods:** The PennPET Explorer is a multiring system designed with a long axial field of view. The imager is scalable and comprises multiple 22.9-cm-long ring segments, each with 18 detector modules based on a commercial digital silicon photomultiplier. A prototype 3-segment imager has been completed and tested with an active 64-cm axial field of view. **Results:** The instrument design is described, and its physical performance measurements are presented. These include sensitivity of 55 kcps/MBq, spatial resolution of 4.0 mm, energy resolution of 12%, timing resolution of 256 ps, and a noise-equivalent count rate above 1,000 kcps beyond 30 kBq/mL. After an evaluation of lesion torso phantoms to characterize quantitative accuracy, human studies were performed on healthy volunteers. **Conclusion:** The physical performance measurements validated the system design and led to high-quality human studies.

In the last 2 decades, commercial PET scanner performance has improved dramatically with CT-based attenuation correction (1), the use of lutetium oxyorthosilicate or lutetium-yttrium oxyorthosilicate scintillators (2,3), time-of-flight reconstruction (4–7), and, most recently, silicon photomultiplier (SiPM)–based time-of-flight detectors (4,8). However, the axial field of view (AFOV) has not grown; it remains 16–26 cm for the newest commercial SiPM-based scanners (9–11). This choice is due mainly to scintillator and SiPM photosensor costs and the prevalence of clinical ^18^F-FDG scanning focused on measuring lesion SUV, typically at 60 min after injection, when the uptake is assumed to be at steady state. The newest commercial instruments perform an ^18^F-FDG whole-body survey (eyes-to-thighs) with excellent diagnostic quality in 10–20 min using bed translation. The motivation for a long-AFOV PET system is to use its high sensitivity to enhance clinical performance and to enable research applications requiring simultaneous measurement of multiple organ systems (12). It is unknown whether such a system would primarily be used clinically to take advantage of high throughput or low-dose imaging or for research with new radiotracers. We developed the PennPET Explorer whole-body imager to support both clinical and research applications.

The performance benefits of a long-AFOV scanner have been simulated (13,14). A few such systems have been built (15–17) but have not transitioned to clinical or research use. That is expected to change with the introduction of the uEXPLORER from United Imaging Healthcare. Developed for the University of California Davis as part of the EXPLORER Consortium (18), the uEXPLORER has a 195-cm AFOV, allowing single-position total-body imaging (19) with high sensitivity from head to foot. The EXPLORER Consortium also supported the development of our system, which we describe as a whole-body imager, since it neither requires multiposition scanning nor covers the total body, as with the United Imaging Healthcare instrument. The general design of the prototype was described earlier (20,21) as a 3-ring-segment, 70-cm-AFOV system. The system is operational with an active 64-cm AFOV, long enough to demonstrate the merits of simultaneous head and torso imaging. A system of this length also has high sensitivity and can perform short scans with high quality. In addition, the prototype configuration has enabled us to address the major technical and scientific challenges of designing a long-AFOV instrument. The challenges include acquiring and processing very high throughput datasets; reconstructing quantitative images with minimal loss of quality from degrading effects, such as axial parallax from oblique lines of response (LORs); and maintaining reliability as ring segments are added. The prototype evaluated here has 3 ring segments, but the design can be expanded easily. We believe that the optimal axial length of a whole-body imager is in the range of 1.0–1.4 m and that the clinical and research benefits will justify the increased system cost and complexity.

The PennPET Explorer is described in the next section, followed by the data acquisition methods. We then present measurements quantifying the physical system performance, along with phantom and initial human images demonstrating its imaging performance.

## MATERIALS AND METHODS

### Design of the PennPET Explorer

The basic building block of the PennPET Explorer is a detector tile of 64 lutetium-yttrium oxyorthosilicate scintillation crystals, which is currently used in the Philips Vereos PET/CT scanner (9). The 8 × 8 array of 3.86 × 3.86 × 19 mm^3^ crystals is coupled to a digital SiPM developed by Philips Digital Photon Counting (22,23). The SiPM sensor tile is fully digital, with 16 individual devices (dies), each generating an independent time stamp. Each die has 4 pixels (2 × 2) allowing a 1:1 crystal–sensor coupling that leads to nearly ideal crystal identification, high count-rate capability, and superior timing resolution. The detector module design allows close ring spacing and operation at 5°C with the first photon trigger level setting (24) to optimize the timing resolution. The entire gantry and its associated electronics are water-cooled; a chiller is used to cool facility water to approximately 0°C to achieve 5°C at the sensor with less than 1°C variation over the full system. Condensation is prevented by infusion of dry purge air to the scanner. In contrast, the same devices in the Philips Vereos PET/CT scanner (9) operate at 18°C and at a higher trigger level.

**FIGURE 1.**
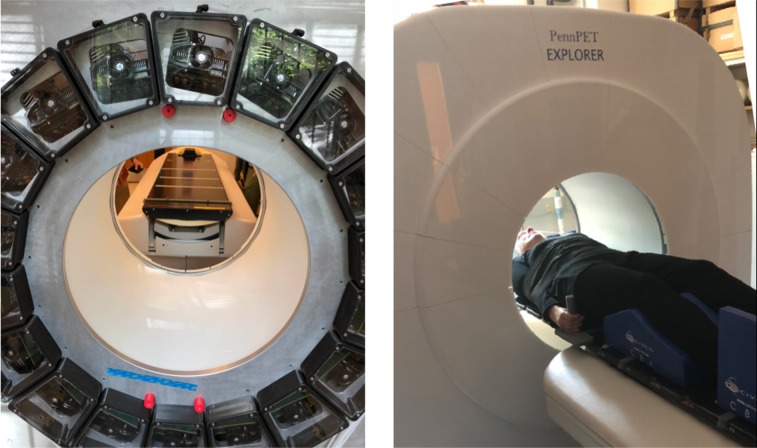
PennPET Explorer in its prototype configuration with 3 ring segments, housed in dry, cool enclosure. View of back of gantry shows modular detector and electronic bays. Also shown is couch with flat pallet installed for human studies.

Each ring segment measures 76.4 cm in diameter and 22.9 cm axially, comprising 18 modules of 28 detector tiles in a 4 × 7 array. The PennPET Explorer is fully sealed and maintains its own cool, dry environment independent of ambient conditions. The ring segments are mounted on linear rails to allow for service access and system expansion. The prototype configuration described in this paper consists of 3 ring segments (Fig. 1).

### Data Acquisition

The data acquisition system uses the Philips Vereos electronic components and collects data as singles events. A master clock distributed to all rings was implemented to eliminate a timing skew between rings. Data (8 bytes per event) are buffered to independent solid-state drive arrays on each ring segment at rates of up to 100 Mcps and then are merged in nearly real-time into a single stream of time-sorted data. Since each ring segment operates autonomously, the system maintains high throughput, independent of the number of ring segments. This results in low dead time, a key requirement for dynamic imaging in which the full tracer bolus will be in the field of view after injection. After transfer from the hardware, the singles data are sorted at a rate of 300 million events/s (with a 16-core Intel Xeon processor) into coincidence list files of prompt and delayed events. The data processing can keep up with acquisition rates even for the most demanding studies, such as a dynamic study after a bolus injection, which has a typical total singles rate of 150 million events/s. Although the singles list file is large, the sorted coincidence list file is 10–20 times smaller and can easily be archived.

### Sensor Calibration

Tile sensors require calibration for consistent energy, timing, crystal, and detector identification encoded for each event. The PennPET Explorer calibrations are based on the calibrations of the Philips Digital Photon Counting tile sensors (22), with run time reduced to about 1 h for parallel (all ring segments) calibration. In brief, the digital SiPM sensor is first calibrated for the photon detection efficiency and timing digital conversion, and an inhibit map is applied to selectively turn off noisy microcells within the digital device. Then, a point source centered in the field of view is used to calculate the energy and timing for each module, and offset values are calculated for each crystal and stored in the module’s on-board memory. The calibrations are stable over many months and are typically needed only after detector module replacement.

Although the data acquisition and calibrations are based on the 16.4-cm AFOV Philips Vereos, we have expanded the axial length of the ring segment to 22.9 cm by adding 2 rows of tile detectors to each module. Read-out of these additional rows required a firmware change that had not been implemented when data were collected for this paper; therefore, these data include gaps of 7.4 cm between the ring segments and an active AFOV of 64 cm rather than 70 cm. The detector geometry is illustrated in Supplemental Figure 1 (supplemental materials are available at http://jnm.snmjournals.org).

### Data Correction and Image Generation

The data correction and image generation methods have been translated from previous work. The scatter correction method was adapted from the time-of-flight–enhanced single scatter simulation method (25,26). With a long-AFOV scanner, the effects of scatter from out-of-field activity are less significant.

We modified the detector efficiency normalization method to accommodate the long AFOV. Previously, a 20-cm-diameter uniform cylinder was used; however, when extended to 1 m or longer, such a phantom is unwieldly and maintaining activity uniformity is difficult. Instead, normalization data are acquired with a thin steel tube filled with approximately 70 MBq of ^18^F centered in a thin-walled, 2.54-cm (1-in)-diameter carbon fiber tube with negligible attenuation that provides rigidity over the 70-cm length. Using an asynchronous motor mounted onto the back plate of the gantry, the line rotates at 2 rpm at a radius just outside the transverse field of view, and data are acquired for about 1 h for an integer number of rotations to collect a sufficient number of counts for all LORs. This method can be extended to a longer AFOV by increasing the carbon fiber tube diameter but maintaining the same wall thickness. Normalization correction factors are generated by calculating the ratio of the collected data to an analytic rotating line sinogram, followed by modest smoothing according to the method of Casey et al. (27).

Phantom and human data were reconstructed using time-of-flight list-mode ordered-subsets expectation maximization (LM-OSEM; 25 subsets, 4 iterations) (28) into 2-mm cubic voxels. This algorithm includes blob basis functions optimized in size and grid spacing for the spatial resolution and noise characteristics of the imager (29). These basis functions suppress image noise while preserving signal; hence, no postfiltering is needed.

Our data format supports a 5-ns maximum coincidence window (8 bits, 19.5-ps time bins), and we set the transverse field of view to 57.6 cm. The PennPET Explorer in its current geometry has a maximum axial acceptance angle of ±40° in the center of the AFOV. The large acceptance angle is not limited in any of the studies presented and leads to the high sensitivity and gain in image signal-to-noise ratio; however, for a typical human body the most oblique LORs are attenuated (30). Thus, for human studies it will be attenuation of the oblique LORs that limits the gain in image signal-to-noise ratio, rather than the axial acceptance angle or the coincidence window.

### Performance Characterization

Performance measurements were taken on the prototype 3-ring-segment system to optimize the hardware and software for acquisition and reconstruction and to demonstrate the capabilities of the PennPET Explorer. The gaps between ring segments halve the sensitivity and reduce the AFOV from 70 to 64 cm. These restrictions did not prevent us from achieving our goal of demonstrating both the technical design and the imaging performance of the whole-body PET imager. For all National Electrical Manufacturers Association (NEMA) performance studies and for phantom and human studies, we used an energy window of 440–660 keV and a coincidence window of 4 ns. All phantom measurements used ^18^F activity, except for the spatial resolution measurements, which used a sealed ^22^Na source.

#### NEMA Measurements

NEMA NU-2 (31) measurements of sensitivity, count-rate performance, timing resolution, and spatial resolution were performed. The sensitivity measurement was performed at 2 positions (radius: 0 and 10 cm) with a 70-cm-long line source inside a set of aluminum tubes. The count-rate measurement was performed with a 70-cm long line source offset inside a 20-cm diameter polyethylene scatter cylinder, at an initial activity concentration of approximately 40 kBq/mL. The count-rate data were also used for measuring the timing resolution as a function of activity. Spatial resolution was measured using a 0.5-mm-diameter ^22^Na point source encased in a 1 cm^3^ plastic cube, imaged at multiple radial positions (1, 5, 10, 15, and 20 cm) and at multiple axial locations (0, 4, 12, 20, 24, and 28 cm) relative to the AFOV center. Per NEMA, single-ring data were reconstructed using the analytic DIRECT algorithm (32). We also report the results from LM-OSEM iterative reconstruction for 1-ring-segment and 3-ring-segment data, using parameters optimized for high-resolution imaging (1 mm^3^ voxels, 4 iterations). Although this may not yield an absolute measure of spatial resolution for a point source in air, the results provide insight into the dependence of spatial resolution on the axial acceptance angle as it increases toward the mid-AFOV.

#### Phantom Measurements

The NEMA image-quality phantom was filled per the harmonization initiative (33). This measurement is similar to NEMA but uses a different body activity concentration and sphere contrast—2 kBq/mL and 9.7:1, respectively. All spheres were hot (per the NU-2 2018 update), with a cold, lunglike region in the center. The NEMA image-quality phantom was positioned with the spheres centered axially. The phantom was imaged with the standard NEMA spheres (diameters of 10, 13, 17, 22, 28, and 37 mm) and with a second set of half-sized spheres (8.5, 11.5, 15, 19, 25, 32, and 44 mm) developed by the harmonization initiative to facilitate matching of the contrast recovery coefficient (CRC) curves between scanners. The phantom was imaged with each set of spheres for 30 min (per harmonization instructions), and the list-mode data were subsampled into scans as short as 1 min. The CRC was calculated using circular regions of interest with diameters equal to the sphere inner diameters, per NEMA NU-2.

The SNMMI Clinical Trials Network lesion phantom (34) has a range of lesion sizes similar to that of the NEMA image-quality phantom, although its 30-cm axial length still falls short of that needed to measure long-AFOV system performance. The phantom was filled with an activity concentration of 5.9 kBq/mL and had a lesion contrast ratio of 4.2:1. The phantom was roughly centered in the AFOV and was imaged for 60 min so the data could be subsampled to shorter scans of 6 min to 15 s, with 10 replicates per scan duration. CRCs were determined for the 10- to 28-mm-diameter spheres.

#### Human Studies

In the evaluation of a new instrument, human studies are crucial to establish the real-world performance beyond phantom studies and to test and optimize data acquisition and reconstruction. A Penn research protocol allows human imaging on the PennPET Explorer. The study has been approved by the University of Pennsylvania Institutional Review Board, and all subjects sign an informed consent form. We have completed 10 human studies, and a more detailed examination of human imaging, including clinical patients, is presented in a companion paper (35). Three studies involving healthy volunteers are shown here: 2 static and 1 dynamic. Subject 1 (female, 62 y old, body mass index of 26.5, 163 cm tall) was injected with 555 MBq of ^18^F-FDG in the clinic, scanned on the clinical Philips Ingenuity TF PET/CT at about 1 h after injection, and then imaged at 1.5 h after injection at a single bed position on the PennPET Explorer for 20 min, the same time as the clinical scan. The full dataset was subsampled and reconstructed to emulate shorter scans, or lower dose. With subject 2 (female, 56 y old, body mass index of 21.6, 155 cm tall), the brain was first imaged near the end of the AFOV and then centered in the AFOV, where the sensitivity is higher but axial parallax errors increase. ^18^F-FDG uptake in the cortical gray matter in the brain provides a good opportunity to evaluate complex structure and the effect of reconstructed spatial resolution. In the third study presented, subject 7 (female, 29 y old, body mass index of 19.3, 177 cm tall) was injected with a fast bolus (∼2 s) inside the PennPET Explorer and imaged for a full hour to capture the early kinetics of ^18^F-FDG for all organ systems simultaneously. Attenuation correction for these studies was done using the CT scan from the Philips Ingenuity TF PET/CT with rigid-body registration (MIM Software, Inc.) applied to the preliminary non–attenuation-corrected PennPET Explorer images. To aid in alignment, affixed to both patient couches was a flat pallet with cushions set with indexing marks so as to position the subjects in a similar manner.

## RESULTS

### Sensitivity

The total sensitivity of the 3-ring-segment PennPET Explorer is 54 kcps/MBq at a radius of 0 cm and 57 kcps/MBq at a radius of 10 cm. Although the sensitivity is much higher than that of commercial instruments, we note that the data gaps in the prototype lead to a 2 times reduction in sensitivity. Normalization correction compensates for these axial sensitivity variations, and the uniformity of a reconstructed long cylinder of activity is demonstrated in Supplemental Figure 1. The excellent image uniformity is a key to achieving quantitative accuracy with phantom and human studies.

### Count Rate

**FIGURE 2.**
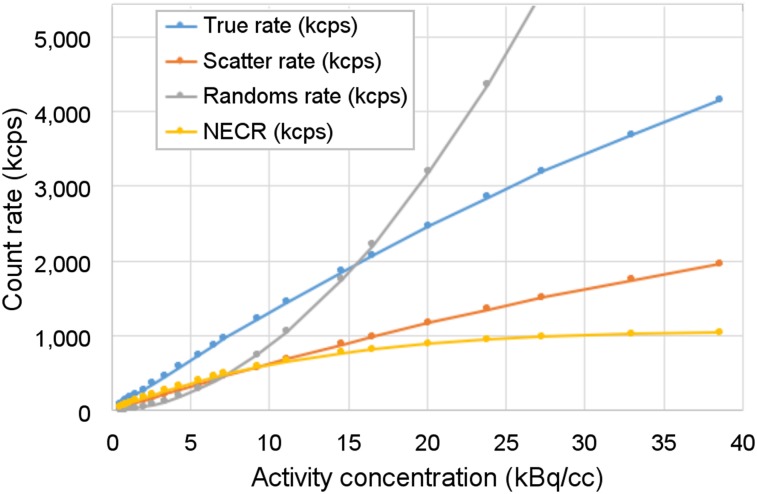
NEMA NU-2 count-rate performance with 70-cm line source inside 20-cm-diameter polyethylene scatter cylinder. Count-rate results were acquired up to 40 kBq/mL, although clinical ^18^F-FDG studies are typically performed with activity concentrations of less than 5 kBq/mL.

The count-rate performance is shown in Figure 2. These results demonstrate that the trues rate is linear over a wide range of activity, up to 38 kBq/mL—about 10 times that of a clinical ^18^F-FDG study. The calculated scatter fraction is 32% and is stable over this range of activity. The noise-equivalent count rate continues to increase slowly beyond the point at which trues equal randoms (at 15 kBq/mL), reaching 1,050 kcps at 38 kBq/mL.

### Timing Resolution

At low activity, the full-system timing resolution is 256 ps, increasing linearly to 284 ps at the highest activity. For individual ring segments, the average timing resolution is 249 ± 6 ps at low activity, increasing to 263 ± 5 ps at the highest activity. Thus, the loss due to oblique LORs in the 3-ring-segment system is modest. There is also close agreement (at low rates) with bench-top timing measurements of 240 ps for individual Philips Digital Photon Counting tiles, implying little loss of performance in translating from single detectors to complete, fully calibrated ring segments.

### Spatial Resolution

Tables 1 and 2 summarize the spatial resolution results, and more detailed plots are shown in Supplemental Figure 2. Transverse results are averages over all axial source positions at that radial position; axial results are averages over all transverse source positions, and the uncertainties shown are the SD over the different source positions. Analytic DIRECT reconstruction was performed with data from 1 ring segment; the spatial resolution (full width at half maximum) in the center of the transverse field of view is about 4.0 mm (transverse and axial), with an expected loss of radial resolution at increased radii. The full width at tenth maximum in the center of the transverse field of view is 8.0–8.5 mm. Spatial resolution results are also shown for LM-OSEM using all data from 1 and 3 ring segments (axial acceptance angle from ±8° to ±40°). Although the absolute values of full width at half maximum can depend on the number of iterations, the LM-OSEM transverse resolution results were similar to those from analytic reconstruction. These results also demonstrate only a small dependence of axial resolution on the axial acceptance angle and on axial location.

**TABLE 1:** Transverse Spatial Resolution of PennPET Explorer Whole-Body Imager

Parameter	Radius (cm)	Rings (*n*)	Algorithm	Radial (mm)	Tangential (mm)	Axial (mm)
FWHM	1	1	Analytic	4.2 ± 0.3	3.9 ± 0.4	4.1 ± 0.2
	1	1	Iterative	3.9 ± 0.3	3.8 ± 0.3	3.6 ± 0.2
	1	3	Iterative	3.9 ± 0.2	3.9 ± 0.3	3.9 ± 0.3
	10	3	Iterative	4.2 ± 0.2	3.9 ± 0.2	3.9 ± 0.3
	20	3	Iterative	5.6 ± 0.2	3.9 ± 0.4	3.7 ± 0.3
FWTM	1	1	Analytic	8.5 ± 0.8	8.4 ± 0.9	7.9 ± 0.2
	1	1	Iterative	7.2 ± 0.4	7.1 ± 0.2	6.8 ± 0.2
	1	3	Iterative	7.4 ± 0.6	7.3 ± 0.2	7.8 ± 1.2
	10	3	Iterative	8.1 ± 0.2	7.2 ± 0.2	7.6 ± 0.8
	20	3	Iterative	10.4 ± 0.3	7.2 ± 0.2	7.3 ± 0.7

FWHM = full width at half maximum; FWTM = full width at tenth maximum.

Uncertainties are SD of replicate measurements.

**TABLE 2:** Axial Spatial Resolution of PennPET Explorer Whole-Body Imager

Parameter	Radius (cm)	Rings (*n*)	Algorithm	Center (mm)	Gap (mm)	Edge (mm)
FWHM	1–20	1	Iterative	—	—	3.5 ± 0.2
	1–20	3	Iterative	4.0 ± 0.2	4.2 ± 0.1	3.6 ± 0.1
FWTM	1–20	1	Iterative	—	—	6.8 ± 0.1
	1–20	3	Iterative	8.5 ± 0.6	8.2 ± 0.1	6.9 ± 0.2

FWHM = full width at half maximum; FWTM = full width at tenth maximum.

Uncertainties are SD of replicate measurements.

### Image-Quality Phantom

**FIGURE 3.**
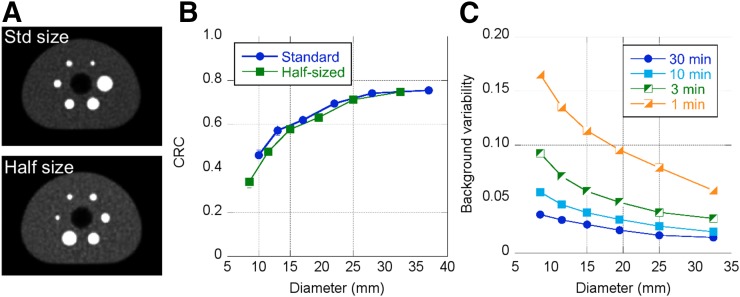
(A) NEMA image-quality phantom shown with standard spheres and half-sized spheres. Body activity concentration is 2 kBq/mL, sphere contrast is 9.7:1, and scan duration is 7.5 min. (B) CRCs as per NEMA guidelines for both standard and half-sized spheres. (C) Background variability as per NEMA guidelines for half-sized spheres.

Images of the NEMA image-quality phantom were acquired sequentially with the standard set of spheres, followed by the half-size set of spheres. In both cases, the spheres were centered axially. The results in Figure 3 demonstrate the value of having smaller spheres where the CRC changes rapidly with diameter and therefore is more sensitive to the choice of reconstruction parameters. Also shown is the background variability for several scan durations. For the current 64-cm AFOV, the updated NEMA prescription suggests a scan duration of about 10 min (scan time = axial step × 30 min/100 cm) using half the AFOV (32 cm) for the axial step to achieve a total-body scan of 100 cm in 30 min. Because the phantom was filled per the harmonization initiative, the activity concentration was about 2 kBq (0.05 μCi)/mL, lower than that prescribed by NEMA (5.2 kBq [0.14 μCi]/mL). Thus, these scan times should be scaled for comparison with NEMA results.

### Clinical Trials Network Lesion Phantom

**FIGURE 4.**
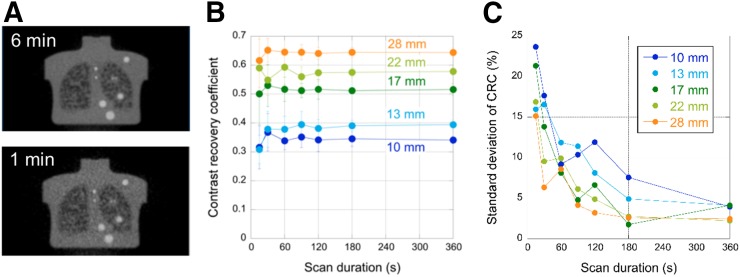
(A) Clinical Trials Network torso phantom with activity concentration of 5.9 kBq/mL and lesion contrast of 4.2:1. (B) CRC of representative lesions as function of scan duration. (C) SD of CRC, determined from replicates of data.

**FIGURE 5.**
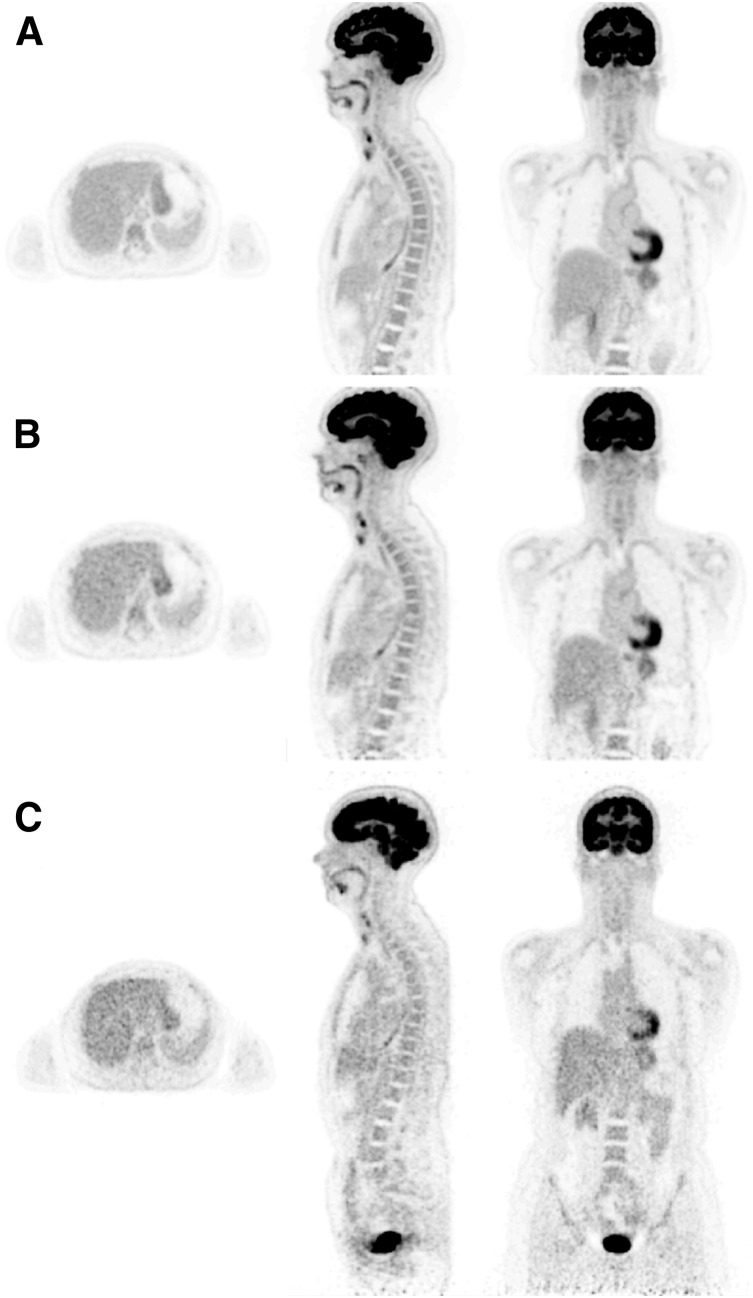
(A) Representative sagittal (left), axial (middle), and coronal (right) views of 20-min PennPET Explorer image of subject 1 at 1.5 h after injection of 555-MBq dose of ^18^F-FDG. All are 2-mm sections. (B) PennPET Explorer image, subsampled (⅛ data) to represent 2.5-min scan. (C) Clinical scan acquired with Philips Ingenuity TF PET/CT scanner at 1 h after injection, using clinical protocol with 10 bed positions for total of 20 min. These data were reconstructed off-line with same reconstruction method as for PennPET Explorer data.

Figure 4A shows typical Clinical Trials Network phantom images for 6- and 1-min acquisitions. CRC as a function of scan duration is shown for different sphere sizes in Figure 4B. CRCs are stable to 60 s (or less), with precision (SD of the CRC mean) of 10% or less for scans shorter than 120 s (Fig. 5C). These results predict good quantitative accuracy for human studies with shorter scan times than used in clinical practice.

### Human Studies

The images from the full 20-min PennPET Explorer scan of subject 1 are shown in Figure 5, along with a PennPET Explorer scan subsampled to represent a 2.5-min scan and the 20-min Ingenuity TF clinical scan (2 min/bed position × 10 bed positions). For this comparison, the clinical data were reprocessed using the same reconstruction tools as the PennPET data, although for routine clinical diagnostics, the clinical data are reconstructed with a smoother basis function and into 4-mm cubic voxels rather than the 2-mm cubic voxels shown here.

**FIGURE 6.**
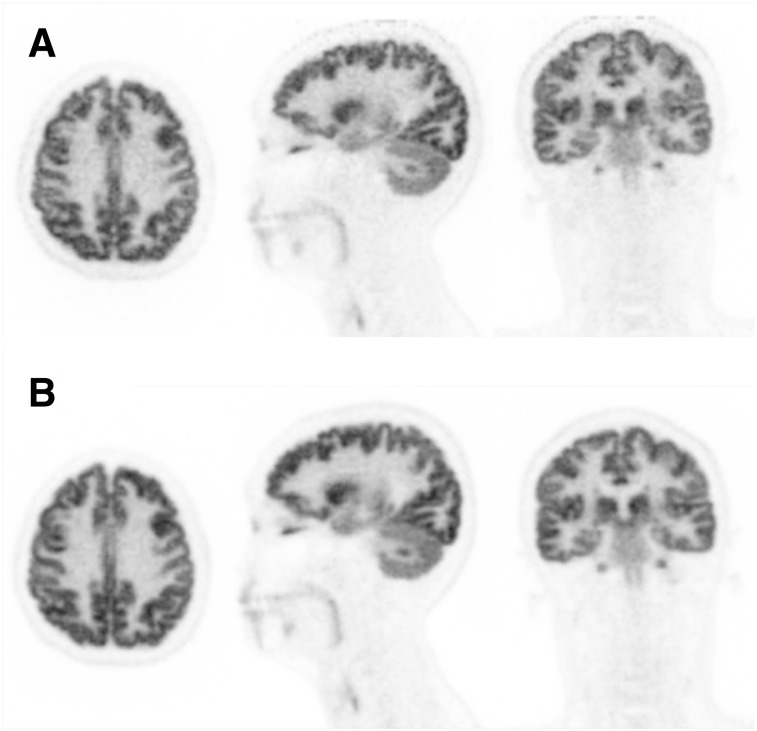
Sagittal (left), axial (middle), and coronal (right) views of PennPET Explorer images of subject 2 positioned with head near edge of AFOV (A) and at center of AFOV (B). These scans were acquired starting at 1.5 h after injection of ^18^F-FDG for 10 min each. All are 1-mm sections.

Figure 6 shows 10-min images of the brain acquired sequentially at 2 axial locations, starting at 1.5 h after injection. Predictably, the image acquired near the edge of the AFOV has approximately 50% of the counts of the image acquired near the center of the AFOV; however, it is difficult to see spatial resolution degradation for the brain in the center of the AFOV despite the larger axial acceptance angle.

**FIGURE 7.**
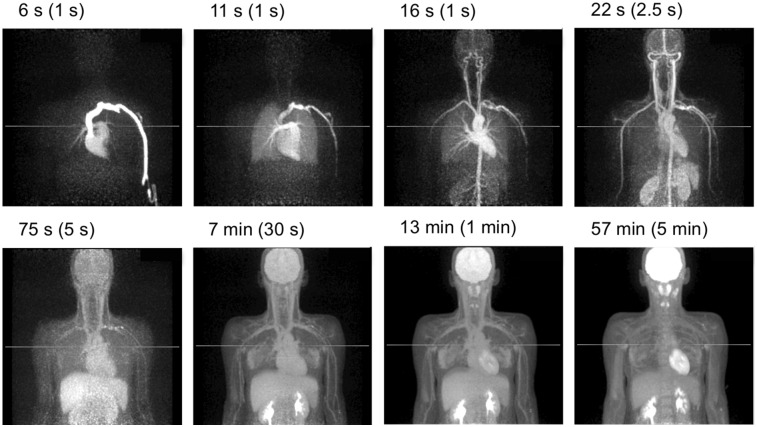
Dynamic ^18^F-FDG study of subject 7 acquired for 60 min after 555-MBq dose of ^18^F-FDG. Representative maximum-intensity projections are shown from 0 to 60 min after injection.

Figure 7 shows representative time frames for subject 7 during the initial uptake, starting with 1-s frames after injection, as well as the final (5-min) frame at 55–60 min. These images demonstrate the enormous potential of dynamic imaging with a large AFOV that includes both brain and torso and with the ability to measure the blood input function and multiorgan kinetics. The singles rate at bolus injection (40 Mcps/ring segment) is in the range of linear behavior and well below our maximum data rate; thus, we can accurately model the tracer behavior.

## DISCUSSION

In this paper, we described the design of the PennPET Explorer and evaluated its performance in its prototype, 3-ring-segment configuration. We focused on tests and metrics that help characterize and illustrate its behavior, particularly in areas that are influenced by the large AFOV compared with existing systems.

We presented a representative set of NEMA tests to characterize the prototype’s performance, including sensitivity, spatial resolution, count rate, and timing resolution measurements. A more complete set of NEMA tests will be performed after the prototype transitions to its final configuration. It is convenient that the prescribed 70-cm-long sensitivity and count-rate phantoms of the NU-2 standard are still applicable for the 64-cm prototype, but some of these measurements may need revision once the imager is extended past 70 cm. The measured sensitivity is very high at 55 kcps/MBq, about 9 times that of a single ring, even with data gaps of about 30% of the ring-segment axial length.

The count-rate capability is typically characterized by the peak noise-equivalent count rate, but we did not reach the peak even at 38 kBq/mL—well beyond expected human protocol rates even with short-lived isotopes (e.g., ^11^C). It may be more relevant to cite the noise-equivalent count rate at a tenth of that activity: 290 kcps at 3.8 kBq/mL. Notably, the true coincident event rate is nearly linear over this wide range of activities, a consequence of the linear single-event rate, which reaches 57 Mcps/ring at 38 kBq/mL.

Point source transverse spatial resolution is 4.0 mm in full width at half maximum near the center (at 1 cm). Radial resolution increases to 5.6 mm at 20 cm. Axial resolution is 4 mm near the center, using data from a single ring segment, but 3-ring-segment data could not be processed with the analytic algorithm because the gaps result in an incomplete dataset. Instead, we demonstrated with the LM-OSEM algorithm that there is only a small loss in axial resolution with 3-ring-segment versus 1-ring-segment data. Care must be taken when iteratively reconstructing point sources in air, but as these results were independent of iteration count (4–10 iterations), we believe they represent the behavior of spatial resolution versus location. We also note that simulations modeling a gapless 3-ring-segment geometry (20,30) predicted an axial resolution loss of 0.5 mm with an analytic algorithm, measured at the AFOV center and including all LORs.

The phantom studies demonstrate the imaging performance of the system and serve as a precursor to human imaging, since they were used to optimize the acquisition and reconstruction choices. The image-quality phantom was imaged per the harmonization initiative, and the CRC values fall in line with those of other modern PET/CT scanners (33) and, importantly, those performed on the Philips Vereos (33), which uses the same underlying hardware. Predictably, our measured noise levels are lower, as expected for a whole-body imager with a long AFOV. The Clinical Trials Network torso phantom was used to better illustrate the behavior of the long-AFOV system, since it is 30 cm in axial length, with lesions throughout the torso region. This phantom demonstrated robust quantitative accuracy for scan durations of 1 min or less, with the precision of the CRC measurement being less than 10% for scans of as short as 2 min.

Performing the first human studies is a critical and exciting step in the development of any new system, as these studies validate its design and operation. Although the whole-body images from the prototype PennPET Explorer have less axial coverage than the typical eyes-to-thighs whole-body survey, these studies represent the quality achievable with a large AFOV. The human images demonstrate image quality superior to that of a clinical standard-of-care ^18^F-FDG scan. By subsampling the list dataset, we have also demonstrated excellent image quality that corresponds to significantly shorter scans or lower dose. We also note that these images, acquired at a single bed position, have uniform noise behavior over the entire AFOV, except at the end slices. This characteristic is important for an instrument designed as an imager rather than a scanner requiring bed translation. The dynamic dataset presents compelling evidence of how such an instrument can be used to probe multiorgan kinetics, particularly as it achieves excellent image quality with time frames as short as 1 s. The progression of the blood and tracer distribution is clearly demonstrated in the first few minutes of the dynamic study. In our companion paper (35), we present additional human studies to explore further new opportunities with a long-AFOV PET imager.

Although the PennPET Explorer was not deliberately designed with axial gaps, the images presented were acquired with data gaps between ring segments. It has been suggested that gaps between rings could extend the AFOV of an imager (36) or allow for imaging during radiation treatment (37). Our results shows excellent image quality and uniform quantitative behavior independent of axial location, suggesting that gaps between ring segments are an effective means of increasing the AFOV for a given number of detectors. This is an important consideration for a whole-body imaging system whose hardware cost is a practical limitation. In addition, system reliability is critical, since a multiring system has many individual components. We have paid considerable attention to the robustness of the design and have developed many useful quality control and detector monitoring aids. Our experience with the prototype after close to a year in operation is that it will be a reliable system even as additional ring segments are incorporated to expand the AFOV of the whole-body imager. Even so, the impact of localized detector failures will be mitigated by the high amount of redundant data due to the large number of LORs.

## CONCLUSION

The 3-ring-segment prototype PennPET Explorer has been completed and tested. The design of the scalable imaging system was described, and the instrument and imaging performance were characterized. Initial phantom and human studies with the PennPET Explorer demonstrate excellent performance and validate the successful implementation of many key components of the design related to the acquisition and reconstruction of large datasets in a multiring configuration. We plan to integrate the prototype with a CT scanner, and the AFOV will be extended by adding ring segments to expand the capabilities of this whole-body imager for both clinical and research applications and to accommodate a wider range of human height.

## DISCLOSURE

This work was supported by NIH R01-CA206187, R33-CA225310, and R01-CA113941 and by the Department of Radiology, University of Pennsylvania, and Philips Healthcare in a research agreement with the University of Pennsylvania. In addition, Amy E. Perkins is an employee of Philips Healthcare. No other potential conflict of interest relevant to this article was reported.

## KEY POINTS

**QUESTION:** How does larger axial coverage of a PET instrument lead to improved image quality and affect quantitative accuracy?
**PERTINENT FINDINGS:** The performance of the prototype configuration of the PennPET Explorer has been tested, and the initial phantom and human studies demonstrate excellent image quality. These studies validate the successful implementation of the key components of the design related to the acquisition and reconstruction of large datasets in a multiring configuration.
**IMPLICATIONS FOR PATIENT CARE:** The high sensitivity and large axial coverage of the PennPET Explorer whole-body imager will lead to benefits for clinical ^18^F-FDG studies and enable translational research that leverages the ability to measure kinetics in multiorgan systems.
