# Supplementary material for: PennPET Explorer: Design and Preliminary Performance of a Whole-Body Imager
Source: J Nucl Med. 2020 Jan;61(1):136–43. doi: 10.2967/jnumed.119.229997 (PMC6954465; doi:10.2967/jnumed.119.229997)
Supplement: Supplementary file 1 [file jnm229997SupplementaryData.pdf]

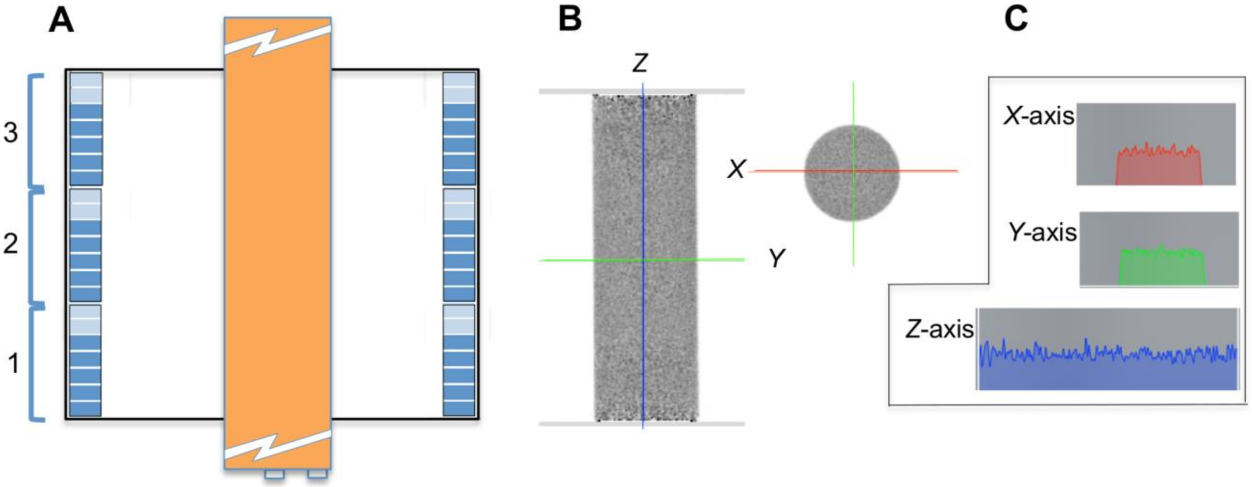

Supplemental FIGURE 1. **A)** Schematic of detector geometry for 3 rings, illustrating the active detector (5 rows, dark blue) and inactive detector (2 rows, light blue) for each ring, leading to gaps in collected data. Illustration includes pipe phantom, 20-cm diameter by 1.2 m long. **B)** Axial (sagittal) and transverse images, each 2-mm thick, following a 1-hr scan with 222 MBq (6 mCi) ^{18}F. **C)** Profiles through images in X-Y (transverse) and Z (axial) directions.

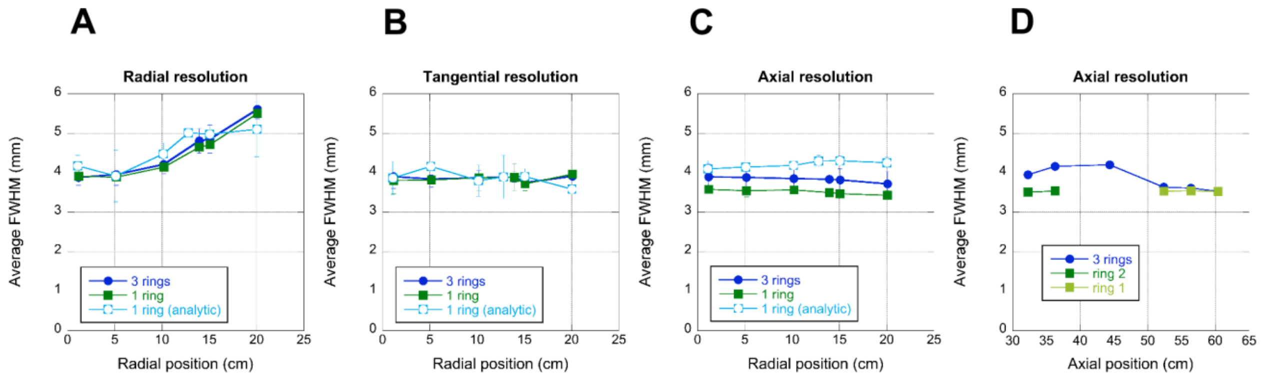

Supplemental FIGURE 2. **A)** Radial spatial resolution with analytic DIRECT and iterative LM-OSEM. Analytic results are shown only for a single ring, whereas LM-OSEM results are shown for data from a single ring and all 3 rings together. **B)** Tangential spatial resolution as a function of radial position. **C)** Axial spatial resolution as a function of radial position. **D)** Axial spatial resolution as a function of axial position. Position 32 cm is at the center of the AFOV, 44 cm is in a gap, and 62 cm is near the edge.
